# Amicoumacin A induces cancer cell death by targeting the eukaryotic ribosome

**DOI:** 10.1038/srep27720

**Published:** 2016-06-14

**Authors:** Irina V. Prokhorova, Kseniya A. Akulich, Desislava S. Makeeva, Ilya A. Osterman, Dmitry A. Skvortsov, Petr V. Sergiev, Olga A. Dontsova, Gulnara Yusupova, Marat M. Yusupov, Sergey E. Dmitriev

**Affiliations:** 1 Institut de Génétique et de Biologie Moléculaire et Cellulaire (IGBMC), INSERM U964, CNRS UMR7104, Université de Strasbourg, 67404, Illkirch, France; 2 Belozersky Institute of Physico-Chemical Biology, Lomonosov Moscow State University, Moscow, 119234 Russia; 3 School of Bioengineering and Bioinformatics, Lomonosov Moscow State University, Moscow, 119234 Russia; 4 Department of Chemistry, Lomonosov Moscow State University, Moscow, 119234 Russia; 5 Engelhardt Institute of Molecular Biology, Russian Academy of Sciences, Moscow, 119991, Russia; 6 Department of Biochemistry, Biological Faculty, Lomonosov Moscow State University, Moscow, 119234 Russia

## Abstract

Amicoumacin A is an antibiotic that was recently shown to target bacterial ribosomes. It affects translocation and provides an additional contact interface between the ribosomal RNA and mRNA. The binding site of amicoumacin A is formed by universally conserved nucleotides of rRNA. In this work, we showed that amicoumacin A inhibits translation in yeast and mammalian systems by affecting translation elongation. We determined the structure of the amicoumacin A complex with yeast ribosomes at a resolution of 3.1  Å. Toxicity measurement demonstrated that human cancer cell lines are more susceptible to the inhibition by this compound as compared to non-cancerous ones. This might be used as a starting point to develop amicoumacin A derivatives with clinical value.

Development of small molecule translation inhibitors is needed for progress in antibacterial as well as anticancer therapy^1^,^2^. Amicoumacin A (Fig. 1a) is an isocoumarin antibiotic that was found among secondary metabolites of a number of soil and marine bacteria^3^,^4^,^5^. Antimicrobial, antiulcer, and anti-inflammatory activity was described for this antibiotic^3^,^4^. The toxicity of amicoumacin A^5^ and closely related compounds^6^ towards cancer cell lines was described, although it was not compared to toxicity for non-cancerous cell lines.

In a recent study^7^, X-ray crystallographic structure of amicoumacin A bound to a *Thermus thermophilus* ribosome as well as biochemical and genetic analysis of bacterial translation inhibition has been reported. It appeared that amicoumacin A binds a conserved site between the E-site mRNA codon and 16S rRNA. The antibiotic contacts only the RNA backbone and nucleobases of rRNA. A number of antibiotics such as pactamycin^7^,^8^, kasugamycin^9^, and edeine^10^ occupy binding sites on the 30S subunit that overlap that of amicoumacin A^7^. All of them either prevent mRNA accommodation in the ribosome or disturb mRNA geometry. In contrast, amicoumacin A mediates additional contacts between the ribosome and mRNA, which may explain its interference with translocation.

The crystal structure of bacterial ribosome in complex with amicoumacin revealed that antibiotic interacts with universally conserved nucleotides of the small subunit rRNA^7^. This suggests that amicoumacin A may also target the eukaryotic ribosome. In support of this assumption, some clinically important effects of the antibiotic on living animals were detected^3^,^5^. However, no direct evidence of its activity in eukaryotic translation systems has been reported.

Although the major principles of protein biosynthesis are uniform in all domains of life, the bacterial and eukaryotic translational machineries substantially differ in some particular components, including ribosome constituent elements^11^,^12^,^13^. The elongation cycle is mostly conserved and assisted by homologous elongation factors^12^, while the difference is notable in translation initiation factors and mechanisms^14^,^15^. Here, we used two evolutionary distant eukaryotic systems (i.e., mammalian and fungal) to assess inhibitory activity of amicoumacin A. We applied *in vitro* translation and mRNA transfection approaches as well as a toe-printing technique to show that amicoumacin A inhibits translation in yeast and mammalian systems by affecting translation elongation. We also compared human cancerous and non-cancerous cell lines for their susceptibility for protein synthesis inhibition by the antibiotic. The structure of the amicoumacin A complex with yeast ribosomes was determined by X-ray crystallography at resolution up to 3.1 Å. While the overall binding site of amicoumacin A in eukaryotic ribosomes appeared to be the same as in bacterial ones, certain differences in the elements of the binding site may provide a framework for designing selective inhibitors on the basis of the amicoumacin A scaffold.

## Results

### Amicoumacin A inhibits mammalian mRNA translation

Mammalian mRNAs are known to utilize a wide spectrum of translation initiation pathways^14^,^16^. This prompted us to start the analysis of amicoumacin A activity in eukaryotes by using mammalian systems. The two most well studied modes of eukaryotic ribosome recruitment are cap-dependent scanning^17^ and viral IRES-mediated initiation^18^. Structural study of the amicoumacin A in the complex with bacterial 70S ribosome showed that inhibitor mediates additional contacts between mRNA and rRNA in the small ribosomal subunit E-site. It could therefore interfere with eukaryotic mRNA translation not only at the elongation step but also during scanning of mRNA leader. Keeping this in mind, we created a set of luciferase mRNA reporter constructs that included both cap-dependent and IRES-dependent transcripts. To exclude possible impact of transcription and other DNA-related events, we took advantage of the mRNA transfection technique^19^.

We prepared polyadenylated transcripts that encoded either *Renilla* luciferase (Rluc) or firefly luciferase (Fluc). The former contained the 5′-untranslated region (5′UTR) of the human β-actin mRNA and were m^7^G-capped. Thus, its translation should reflect a behaviour of a regular cellular mRNA. In contrast, each of the Fluc encoding transcripts harboured one of three well-characterized viral IRESs in their 5′UTR. We used IRES elements that differ in translation initiation mechanisms and have different initiation factor (eIF) requirements. Encephalomyocarditis (EMCV) IRES requires the same set of eIFs as the Actin-Rluc, except for the cap-binding protein eIF4E; it places the 40S ribosome subunit into its internal region, so there is only a limited scanning event across a few nucleotide-long initiation window in this case^18^. Hepatitis C virus (HCV) IRES binds the 40S directly and loads it precisely onto its AUG start codon; thus, it does not require any mRNA-binding eIF4 factors, and it is also, under some conditions, able to initiate translation *via* eIF2-independent pathways^18^. Cricket paralysis virus (CrPV) IRES does not need initiation factors or Met-tRNA_i_ at all, since it utilizes the most exotic initiation mechanism ever^18^. Thus, in the two last cases, there is not even a limited scanning during translation initiation.

**Figure 1:**
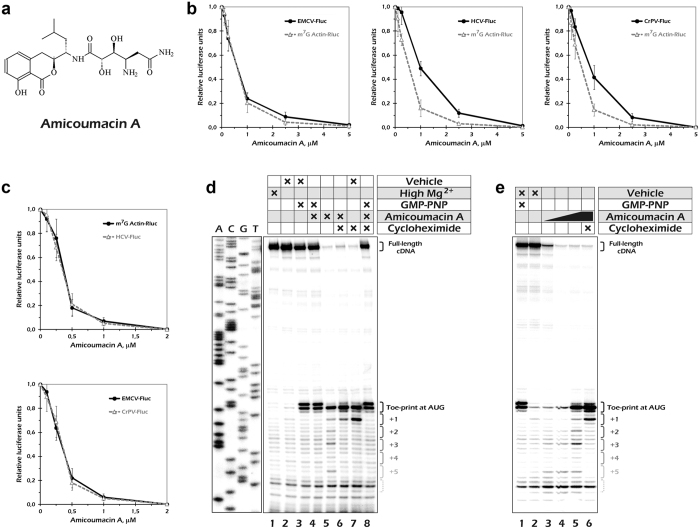
Amicoumacin A inhibits mammalian mRNA translation. (**a**) Chemical structure of amicoumacin A. (**b**) Inhibition of reporter mRNA translation by amicoumacin A in HEK293T cells. Error bars represent the standard deviations of the mean values for at least three independent experiments. (**c**) Inhibition of reporter mRNA translation by amicoumacin A in Krebs-2 cells S30 extract. (**d)** Ribosome stalling by amicoumacin A and other antibiotics in rabbit reticulocyte lysate as revealed by toe-printing assay. Cross signs denote components added to the reaction mixture. Final concentrations of the additives were as follows: 15 mM Mg(OAc)_2_ (lane 1); 0.2% EtOH (lane 2); 2 mM GMPPNP and 0.2% EtOH (lane 3); 2 mM GMPPNP and 100 μM amicoumacin A (lane 4); 100 μM amicoumacin A (lane 5); 100 μM amicoumacin A and 1 mM cycloheximide (lane 6); 1 mM cycloheximide and 0.2% EtOH (lane 7); 2 mM GMPPNP, 100 μM amicoumacin A and 1 mM cycloheximide (lane 8). Note that the toe-print pattern produced by the 48S complex (lanes 3, 4 and 8) differs from that made by the elongating 80S ribosome (lanes 5–7), in accordance with the previous observation^23^. (**e**) Inhibition of ribosome movement along mRNA by amicoumacin A. The antibiotic concentrations were 0, 1, 10 or 100 μM (in lanes 1–2, 3, 4 and 5–6, respectively).

We transfected the reporter mRNA constructs into cultured HEK293T cells and measured the luciferase activity after 2 hours of expression. Increasing concentrations of Amicoumacin A were added to the culture medium just before the transfection. The short incubation period allowed us to minimize secondary effects that could appear if prolonged incubations with the drug were used. Translation of both scanning- and IRES-dependent mRNAs were inhibited by amicoumacin A in a micromolar concentration range (Fig. 1b). Almost identical inhibition curves were obtained for Actin-Rluc and EMCV-Fluc mRNAs, while translation of both HCV-Fluc and CrPV-Fluc were slightly more resistant to the drug.

The difference we observed could be explained by either an additional inhibitory action of the antibiotic on the scanning process or by some indirect effects of the drug. It is long time known that some protein synthesis inhibitors cause ribotoxic stress response in mammalian cells, including eIF2 phosphorylation and partial inactivation^20^,^21^. In agreement with the latter, we detected a slight increase in eIF2α-P level in treated cells (Supplementary Fig.1). To investigate this further, we performed an *in vitro* translation experiment in the mammalian cell-free system that closely recapitulates *in vivo* conditions^22^. We did not observe significant differences between scanning- and IRES-dependent translation in this system (Fig. 1c). Thus, the effect of amicoumacin A on mammalian mRNA translation is most likely limited to the elongation stage and does not depend on initiation mode.

To dissect this issue unambiguously, we monitored ribosome progression along the capped β-globin mRNA by toe-printing in rabbit reticulocyte lysate^23^. We observed a clear toe-print band corresponding to a ribosome at the AUG codon (Fig. 1d). The pattern of the toe-print from amicoumacin A arrested ribosomes, the single band at position +17 relative to the AUG (Fig. 1a, lane 5), matched that of the 80S particles^23^. There were also a few minor bands with a three-nucleotide periodicity (Fig. 1a, lane 5) that probably reflected positions of ribosomes that avoided antibiotic-mediated arrest at the AUG. Importantly, we did not detect any antibiotic-dependent bands at the upper part of the gel (besides the full-length cDNA signal), which could be interpreted as toe-prints from the stalled scanning complexes. In agreement with this, there was no amicoumacin-induced difference in signal intensity of 48S complex toe-prints obtained at the AUG codon with GMPPNP (Fig.1d, lanes 3 and 4). It should be noted that pronounced ribosome stalling at the AUG could be observed only at rather high antibiotic concentration (100 μM), while lower concentrations also caused a gradual decrease in the full-length cDNA signal intensity (Fig. 1e). Obviously, under these concentrations the antibiotic was unable to arrest the majority of the elongating ribosomes during the first elongation step and generated a series of ribosomal complexes halted at different positions on the mRNA that could not be visualized as distinct toe-print bands. In summary, these results clearly showed that amicoumacin A inhibits translation elongation in mammalian systems.

### Amicoumacin A inhibits translation in yeast

**Figure 2:**
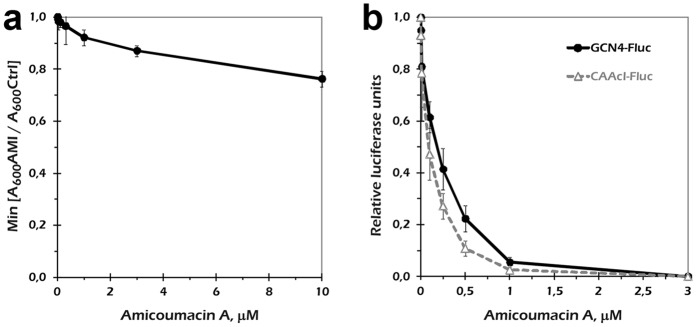
Amicoumacin A action on yeast *S. cerevisiae*. (**a**) Yeast culture growth in the presence of amicoumacin A. OD600 values were divided to that in a plate well without the drug and taken at the time point of 11 h (when these ratios were minimal, as shown in Supplementary Fig. 2). The maximum concentration of the drug used in this experiment (10 μM) was equal to 4.2 μg/ml amicoumacin A added into the medium. Error bars represent the standard deviations of the mean values for three replicates. (**b**) Inhibition of reporter mRNA *in vitro* translation by amicoumacin A in the yeast cell-free system. Error bars represent the standard deviations of the mean values for three independent experiments.

Another eukaryotic system that is widely used for analysis of translation inhibitors is budding yeast. We monitored yeast culture growth in the presence of increasing amicoumacin A concentrations. However, we did not observe substantial growth inhibition up to the highest antibiotic concentration we used (Fig. 2a, Supplementary Fig. 2). We hypothesized that this tolerance could be due to one or more of the following: a limited amicoumacin A penetration through the yeast cell wall, its efficient removal out of the cell, or metabolization of the drug by living yeast cells.

To exclude any effect not related to translation, we performed an *in vitro* experiment in yeast cell-free system (Fig. 2b). Two luciferase-encoding transcripts with either artificial leader (CAA_19_cI-Fluc) or a 5′ UTR from the natural yeast GCN4 gene (GCN4-Fluc) were used. Both mRNAs initiate translation by canonical scanning mechanism, although the latter one bears four uORFs in their leader and is a subject of a eIF2-P-mediated translational control^17^. Translation of both mRNAs was inhibited by increasing concentrations of amicoumacin A, and the effect was slightly higher for CAA_19_cI-Fluc. This result indicates that cell integrity protects yeast ribosomes from amicoumacin A inhibition *in vivo*, while the ribosomes themselves are highly susceptible to inhibition.

### Structure of the amicoumacin A complex with yeast ribosome

**Figure 3:**
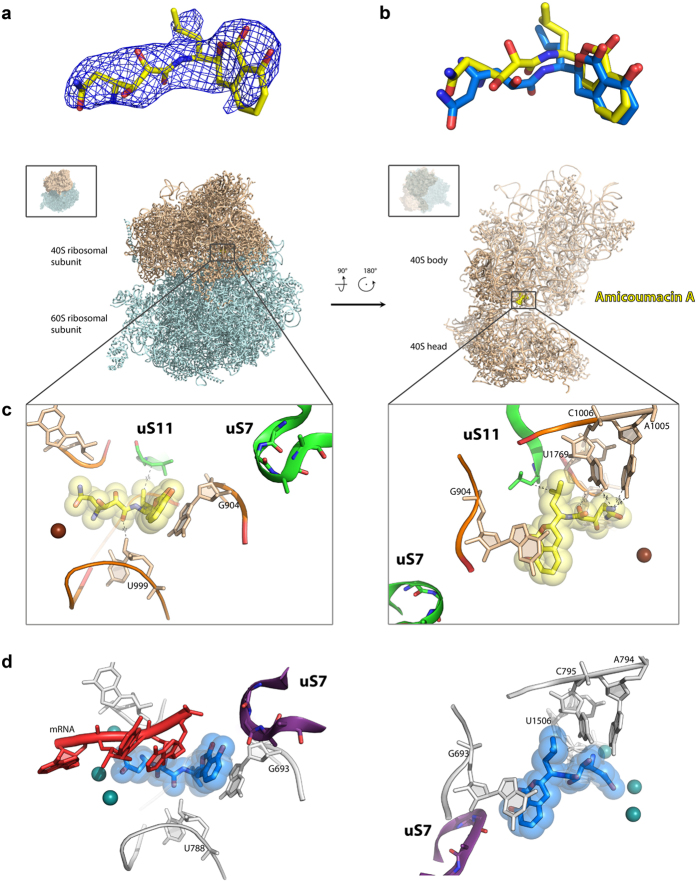
Structure of the amicoumacin A complex with yeast ribosome. (**a**) Difference electron density map of amicoumacin A in complex with 80S ribosome. The map is contoured at 2σ. Amicoumacin A is shown in yellow. (**b**) Comparison of amicoumacin A conformation in yeast 80S and bacterial 70S ribosome. The 70S ribosome from *T. thermophilus* (PDB entry 4W2F) in complex with amicoumacin A (shown in blue) was aligned on the 80S ribosome based on the helix 23 in 18S or 16S rRNA (ribosomal components are omitted for clarity). (**c**) The binding pocket of amicoumacin A in small ribosomal subunit E-site. Two orientations of the 80S ribosome in complex with amicoumacin A are shown in the upper panel. The view from the side of the 40S head is shown on the left, and the view from the subunit interface is shown on the right (60S subunit is omitted for clarity). The 40S subunit is colored in wheat, and the 60S subunit is in light blue. The binding pocket of amicoumacin A is magnified in the lower panel. Amicoumacin A is shown in yellow, rRNA residues (in wheat, proteins uS11 and uS7) in green, and magnesium ions in brown. Interactions of amicoumacin A are depicted with the dashed lines. (**d**) Comparison of the amicoumacin A binding site in a yeast 80S ribosome with the one in bacterial 70S ribosome from *T. thermophilus*. Amicoumacin A is shown in blue, rRNA residues in grey, protein uS7 in violet, and magnesium ions in dark green (PDB entry 4W2F).

The ability of amicoumacin A to inhibit yeast ribosomes prompted us to use this system for structural study. For this, we applied the procedure to determine the structures of 80S ribosome from *Saccharomyces cerevisiae* in complex with translation inhibitors^24^. We prepared the crystals of 80S ribosome as described and introduced amicoumacin A by soaking at concentration 200 μM during post-crystallization treatment. The dataset was collected from a single crystal, and data with a maximum resolution of 3.1 Å were included for refinement. An initial unbiased difference electron density map (F_obs_ − F_calc_) was calculated using the model of vacant 80S yeast ribosome (PDB entry 4V88). The amicoumacin A molecule was located in the E-site of small ribosomal subunit after manual inspection of the peaks of positive electron density (Fig. 3a). The atomic model of amicoumacin A and its geometry restraints were generated with the help of a Grade web server (Global Phasing, http://grade.globalphasing.org). The structure of 80S-amicoumacin A complex was refined with Phenix.refine^25^. Final statistics of the data collection and refinement are shown in Table 1.

Amicoumacin A interacts mostly with rRNA residues in helixes 23 and 24 (Fig. 3c). It is involved in stacking with G904 (G693 in bacteria) in the tip of h 23. The antibiotic is located within the hydrogen bond distance from 2′-OH of ribose of U999 (U788), oxygen of the phosphate group of U1769 (U1506), and from Watson-Crick edges of nucleotides A1005 (A794) and C1006 (C795).

We compared the structure of amicoumacin A bound to the yeast ribosome to the structure of the bacterial ribosome from *Thermus thermophilus* in complex with amicoumacin A reported previously^7^ (Fig. 3b–d). We did not observe the ions coordinated by amicoumacin A in contrast to the structure with bacterial ribosome that can be attributed to the lower resolution of the obtained data (3.1 Å here compared to 2.4 Å in^7^). Otherwise, the conformation adopted by amicoumacin A in bacterial and in eukaryotic ribosomes is very similar. A small shift in the position of 2,3-dihydroxy-5-aminohexandiamide tail can be attributed to the absence of mRNA in the present model. Remarkably, the contacts of amicoumacin A with the eukaryotic ribosome are preserved in the absence of functional ligands.

The differences in the binding site of amicoumacin A in bacterial and yeast ribosomes^7^ are found in the proteins uS11 and uS7 (Fig. 3c,d). The loop of uS7 in a bacterial ribosome is located within 3.9 Å from the isocoumarin moiety of amicoumacin A and is more than 10 Å away in the eukaryotic ribosome. The C-terminal tail of uS11 contains an eukaryote-specific extension and is oriented differently in the 80S ribosome. It does not reach the amicoumacin A binding pocket in the *Thermus thermophilus* 70S ribosome, while in the yeast 80S ribosome the terminal leucine of uS11 forms a hydrophobic interaction with the isobutyl group of amicoumacin A. The mutations in the C-terminus of uS11 are known to confer resistance to cryptopleurine and emetine^26^,^27^. Cryptopleurine and emetine along with pactamycin bind small subunit E-site in a similar way making strong stacking interactions with G904^24^,^28^. On the basis of the structure, we can hypothesize that these mutations would also lead to resistance of the yeast translation to amicoumacin A *in vitro*. Interestingly, cryptopleurine and emetine are active only in eukaryotes, while pactamycin is a universal inhibitor.

### Amicoumacin A preferentially inhibits cancerous cell lines

Cancerous transformation usually is accompanied by hyperactivation of translation machinery^29^,^30^. High demand for protein biosynthesis makes cancerous cells more sensitive to inhibitors of translation than normal cells. In order to evaluate a potential of amicoumacin A as a possible anti-cancer compound, we determined its toxicity towards the MCF-7 breast cancer cells, the A549 lung cancer cells, and two cell lines of non-cancerous etiology (HEK293T embryonic kidney and VA13 lung fibroblast cell lines), using the MTT assay (Table 2). The concentrations of amicoumacin A that caused 50% growth inhibition or cell death (IC_50_) roughly matched that of inhibition of reporter mRNA translation (Fig. 1). This argues for the idea that translation is the primary target of amicoumacin A in mammalian cells. As expected, cancer cells appeared to be 2–4 times more susceptible to amicoumacin A inhibition than the cell of non-cancerous etiology. There were also a correlation between IC_50_ and the cell culture growth rates that were decreased in the raw: A549≈HEK293T > MCF7 ≫ VA13. Although selectivity is not high, it may lay the basis for further work on improvement of amicoumacin A properties as an anti-cancer chemical.

## Discussion

Ribosomes are one of the most conserved molecular assemblies, and at the same time they are the target of many antibiotics. A large cohort of protein synthesis inhibitors affects both bacterial and eukaryotic ribosomes^1^,^24^. As a rule, these antibiotics bind to the conserved sites of the ribosome. In an earlier report, amicoumacin A was demonstrated to inhibit bacterial ribosomes by providing additional interactions between mRNA and rRNA^7^. Movement of mRNA was suggested to be inhibited by amicoumacin A.

In eukaryotes, ribosome movement along mRNA occurs not only during elongation but also upon the initiation step of protein synthesis. Mammalian cells utilize diverse mechanisms of interaction with mRNA during translation initiation^14^,^16^. In the canonical pathway, small ribosomal subunit moves along 5′UTR searching for a start codon^17^. In contrast, some viruses employ internal ribosome binding using a variable set of initiation factors depending on their IRES type^18^. In this work, we demonstrated that amicoumacin A does not affect any mode of translation initiation that we have tested. In contrast, it inhibits eukaryotic ribosome movement along mRNA during the elongation stage of protein synthesis in a similar way as it does with bacterial ribosome. The only effect of amicoumacin A on translation initiation is the slight phosphorylation of eIF2 due to induction of stress response.

Inhibition of the eukaryotic protein synthesis may be employed for the development of anti-cancer or immunosuppressive agents^2^. In 2012, the first protein synthesis inhibitor targeting the eukaryotic ribosome, omacetaxine mepesuccinte (homoharringtonine), was approved by the FDA for treatment of chronic myeloid leukemia^31^. Recently, a number of well-known translation inhibitors were shown to possess high activity against breast cancer cells^32^. In line with this, we demonstrate here that toxicity of amicoumacin A for cancer cell lines is several times higher than for non-cancerous cell lines.

Amicoumacin A is the universal translation inhibitor since its binding pocket in the E-site of the small ribosomal subunit is highly conserved (Fig. 3). It interacts either with universally conserved rRNA residues or with a backbone of rRNA and mRNA in a sequence-independent manner. Comparison of the structures of amicoumacin A complexes with bacterial^7^ and eukaryotic ribosomes paves the way to the development of derivatives that may have better selectivity. While the RNA elements of the amicoumacin A binding site are absolutely identical for bacterial and eukaryotic ribosomes, structures of ribosomal proteins surrounding amicoumacin A on the ribosome are different (Supplementary Fig. 3). C-terminal amino acid of yeast ribosomal protein uS11 is located at 3.6 Å from the isobutyl group of amicoumacin A. Mutations of uS11 make yeast ribosomes resistant to the inhibition by cryptopleurine and emetine^26^. These translation inhibitors bind a site overlapping that of amicoumacin A^24^. C-terminus of uS11 is not well resolved in the structure of bacterial ribosomes from *Thermus thermophilus* in complex with amicoumacin A (Supplementary Fig. 3a). However, more detailed analysis and comparison with the structure of uS11 in the *Escherichia coli* ribosome reveals a 1 Å shift of Cα of the C-terminal amino acid interacting with isobutyl group of amicoumacin A (Supplementary Fig. 3b)^33^. Moreover, this amino acid is a valine in *E. coli* and leucine in *S. cerevisiae*.

A conserved loop of the ribosomal protein uS7 is located 3.9 Å from the isocoumarin part of amicoumacin A in the bacterial ribosome and is more than 10 Å away in the yeast 80S structure (Supplementary Fig. 3c). Large deviations can be attributed to the different states of the ribosome in two structures (classical in *T. thermophilus* compared to ratcheted in *S. cerevisiae*). Since this loop has a special importance in the process of start codon selection during translation initiation^34^, we focused our attention on this particular structural element. We aligned uS7 from the structure of a yeast ribosome to the uS7 in bacterial ribosome to model its possible orientation to amicoumacin A (Supplementary Fig. 3d). The alignment shows that this loop can reach the amicoumacin A binding site of yeast ribosomes but adopts different conformation compared to bacterial ribosomes. Altogether, derivation of the isocoumarin part and the isobutyl moiety of the amicoumacin A scaffold might increase selectivity of the compound towards bacterial or eukaryotic ribosomes and may provide new properties to the drug with respect to a modulation of the start codon selection process.

Another intriguing possibility is using the amicoumacin scaffold for designing mRNA-specific translation inhibitors. For example, the HCV IRES domain II binds in close proximity^35^,^36^ to the amicoumacin A binding site on the ribosome (Supplementary Fig. 4). Although the original drug did not show any preferential inhibition of the HCV IRES directed translation (Fig. 1b,c), one may suggest that its derivatives could interfere specifically with the IRES domain II placement onto the ribosomal E-site.

In summary, we presented evidence for amicoumacin A activity toward the eukaryotic ribosome in both mammalian and yeast systems, we revealed structural details of its interaction with the yeast 80S ribosome, and we showed a relative selectivity of the drug toward human cancer cell lines. Our study could be used for rational drug design aiming to improve amicoumacin A therapeutic potential.

## Methods

### Reagents

Amicoumacin A isolation was described earlier^7^. The purified antibiotic was dissolved in ethanol to concentration of 2.5 mM.

### Plasmid constructs and *in vitro* transcription

The plasmids pbG coded for the rabbit β-globin mRNA^37^, pActin-Rluc^38^, pActin-Fluc, and pEMCV-Fluc^22^ were described earlier. Modified pHCV-Fluc with a complete HCV IRES and a single AUG codon in the initiation region was a gift from I. Terenin. To obtain CrPV IRES cDNA, PCR with a plasmid gifted by A. Komar was used with primers GGCGCACTAGTCAGCTGAAAGCAAAAATGTGATCTTGCTTG and СGCCGGCGCCGGGCCTTTCTTTATGTTTTTGGCGTCAAGCTTATTTTCTTGTTTATCTTGAAATGTAGCAGGTAAATT. The PCR product was then treated with PvuII and NarI and inserted into the pEMCV-Fluc plasmid digested by the same enzymes, resulting in the plasmid pCrPV-Fluc. Prior to *in vitro* transcription, pbG was linearized at the EcoRI site. For synthesis of the polyadenylated mRNAs encoding the firefly and *Renilla* luciferases, a 50T-tailed PCR product was used as a template, as described previously^22^,^38^,^39^. In the case of the CrPV-Fluc, the template was generated with CGCCGTAATACGACTCACTATAGGGAAAGCAAAAATGTGATCTTGCTTG as the forward primer. For the transcription, the RiboMAX kit (Promega) was used. The resulting transcripts were precipitated with 2M LiCl. The IRES containing mRNAs were uncapped, whereas for all other transcripts, Vaccinia Capping System (NEB) was used to obtain 100% capped products.

### Toe-printing of ribosomal complexes in rabbit reticulocyte lysate

Ribosomal complex assembly was performed in the nuclease treated RRL (Promega, L4960), as described previously^40^. Briefly, the reaction was initiated in a total volume of 9 μl containing 7 μl of RRL, 2 u of RiboLock RNase inhibitor (Thermo Scientific), and 2 μl of water solution of either an antibiotic, 10 mM GMPPNP·Mg, or 75 mM of Mg(OAc)_2_, as indicated. The mixture was incubated for 5 min at 30 °C, then 1 μl of mRNA solution (0.5 pmol/μl) was added followed by incubation for an additional 10 min. After that, 10 μl of RT Mix (including [^32^P]-labeled primer TCACCACCAACTTCTTCCAC) was added, according to^40^. The mixture was incubated for 20 min at 30 °C. The resulting cDNAs were then purified by thorough phenol/chloroform extraction, precipitated with ethanol, and analyzed on 6% sequencing gel along with a sequence ladder obtained from the corresponding plasmid with the same primer and the Sequenase 2.0 DNA sequencing kit (USB/Affymetrix). Radioactive bands in the dried gels were visualized using the Typhoon FLA 9500 Phosphorimager (GE Healthcare Life Sciences).

### Mammalian cell growth and mRNA transfection

HEK293T cells were cultured and transferred into 24-well plates 12–16 h before transfection, as described^39^. The transfection was performed using Unifectin-56 (Unifect Group, Russia). The standard protocol was modified to obtain maximal mRNA transfection efficiency according to^41^. Amicoumacin A ethanol stock was diluted with water to obtain 100x solutions, as indicated, and was added to the medium right before addition of the transfection complexes. All manipulations were performed in such a way to minimize time of holding the cells out of CO_2_ box and to avoid cooling the plate. Two hours after the transfection, cells were harvested, and luciferase activities were analyzed with the Dual Luciferase Assay kit (Promega). All the transfections were repeated at least three times in different cell passages.

### Yeast strain and growth conditions

The BY4741 (*MATa his3*Δ*1 leu2*Δ*0 met15*Δ*0 ura3*Δ*0*) yeast strain was grown in a YPD medium (2% glucose, 2% bacto-peptone, 1% yeast extract) to exponential phase and inoculated into liquid YPD at OD600 of 0.05. Growth rates (OD600) were measured every 5 min in a 24-well plate in the Infinite 200 PRO microplate reader (Tecan Trading AG, Switzerland) at 30 °C with continuous shaking.

### Mammalian and yeast cell-free systems and *in vitro* translation assays

Krebs-2 ascite cells S30 extract was prepared as described previously^22^. Yeast cell-free extract was prepared according to^42^ with the following changes: homogenated cells were centrifugated once for 15 min at 20000 g, and chromatographic fractionation and nuclease treatment stages were omitted.

Translation experiments in the mammalian system were performed in a total volume of 10 μl, which contained 5 μl of the S30 extract, translation buffer (20 mM Hepes-KOH pH 7.6, 1 mM DTT, 0.5 mM spermidine-HCl, 0.8 mM Mg(OAc)_2_, 8 mM creatine phosphate, 1 mM ATP, 0.2 mM GTP, 120 mM KOAc, and 25 μM of each amino acid), 2 u of RiboLock RNase inhibitor (Thermo Scientific), 0.5 mM D-luciferin, 0.25 pmol mRNA, and 1 μl of amicoumacin A solution, as indicated. Translation reactions in the yeast system were performed in a total volume of 15 μl, containing 7.5 μl of the extract, translation buffer (25 mM Hepes-KOH pH 7.4, 2 mM DTT, 3 mM Mg(OAc)_2_, 12 mM creatine phosphate, 1 mM ATP, 0.4 mM GTP, 126 mM KOAc, and 50 μM of each amino acid), 3 u of RiboLock RNase inhibitor, 50 μg/ml creatine phosphokinase, 1 mM D-luciferin, 0.12 pmol mRNA, and 1 μl of amicoumacin A solution, as indicated. Translation mixtures were incubated in a white 384-well plate (F-bottom, non-binding polystyrol, Grenier GR-781904) and covered by a PCR plate seal at 30 °C (for the mammalian system) or 25 °C (for the yeast one) in the TECAN reader with continuous measurement of the luciferase activity (integration time 3 s). Light intensities at 25 min were taken as luciferase activity values.

### Ribosome complex crystallization, structure determination, and analysis

80S ribosomes from the yeast *S. cerevisiae* were purified and crystallized, as previously described^43^. The 80S ribosome complex with Amicoumacin A was formed by soaking 80S ribosome crystals with 0.2 mM of antibiotic for 2 h at 4 °C during post-crystallization treatment. A single crystal was used for data collection at the SOLEIL synchrotron with the beam line PROXIMA1. We attenuated the beam of the incoming photon flux to collect redundant data in 4 datasets of 90° that were subsequently merged together. Diffraction data were reduced using the XDS suite^44^.

The structure was solved by rigid body refinement of the deposited 80S ribosome structure (PDB 4V88) using Phenix.refine^25^. Electron density maps were inspected manually. Peaks of positive electron density were observed in both ribosomes from the asymmetric unit, but the quality of the density allowed us to model the antibiotic only in one ribosome. Coordinates and restraints for amicoumacin A were generated online with the Grade web server (Global Phasing, http://grade.globalphasing.org) using SMILES strings from the PubChem database^45^. Ligand fitting and remodelling of ribosomal binding sites were performed manually using Coot^46^. Final refinement of atomic coordinates, atomic displacement parameters, and occupancies was performed with Phenix.refine. Crystallographic statistics are reported in Table 1. Figures of structures were prepared using PyMOL 1.5 (Schrödinger, http://pymol.org/). Ribosomal proteins were named throughout the manuscript according to the newly established nomenclature^47^. Atomic coordinated and structure factors for the 80S-amicoumacin A structure have been deposited in the Protein Data Bank (http://pdb.org/pdb/home/home.do) under accession code 5I4L.

### MTT assay

Cytotoxicity was assessed using the MTT (3-(4,5-dimethylthiazol-2-yl)2,5-diphenyl tetrazolium bromide) assay based on the method described by Ferrari and co-workers^48^, with some modifications. 4000 cells per well for VA-13 cell line and 3000 cells per well for MCF7, HEK293T and A549 cell lines were plated out in 135 μl of DMEM/F12 media in a 96-well plate and incubated at 37 °C, 5% CO_2_ for 18 h before treatment. Then we added 15 μl of the drug (as a media/DMSO solution, the final DMSO concentrations in the media were 1%) and incubated the cells for additional 72 h. Amicoumacin A in final concentrations of 50 nM–100 μM (eight dilutions), in triplicate, was applied. 2 nM–6 μM doxorubicin was used as a positive control. At the end of the incubation we added MTT into the media (up to 0.5 mg/ml), incubated the cells for 2 h, followed by removing the media and addition of 100 μl DMSO. The amount of MTT reduced by cells to its blue formazan derivative was measured spectrophotometrically at 565 nM using a plate reader and normalized to the values for cells treated with the media/DMSO only. IC50 was calculated with “GraphPad Prism 5” software (GraphPad Software, Inc., San Diego, CA).

## Additional Information

**How to cite this article**: Prokhorova, I. V. *et al*. Amicoumacin A induces cancer cell death by targeting the eukaryotic ribosome. *Sci. Rep*. **6**, 27720; doi: 10.1038/srep27720 (2016).

## Supplementary Material

Supplementary Information

## Figures and Tables

**Table 1:** Data collection and refinement statistics.

Space group	P2_1_
Data collection	
Cell dimensions	
a, b, c (Å)	434.23 287.91 304.12
α, β, γ (°)	90 99.11 90
Resolution (Å)	103.62-3.10 (3.20–3.10)
R_meas_**	22.80 (159.10)
I/σI	6.92 (1.02)
CC_1/2_	99.00 (44.20)
Completeness (%)	99.97 (100.00)
Redundancy	6.64 (5.11)
Refinement	
Resolution (Å)	103.619–3.100
No. unique reflections	1329824
R_work_/R_free_	0.2009/0.2478
Total No. atoms	410489
Average B-factor	79.520
R.m.s deviations	
Bond lengths (Å)	0.009
Bond angles (°)	1.356

**Table 2:** Toxicity of amicoumacin A to human cell lines.

Cell line	C/NC*	IC_50_, μM (IC_50_, μg/ml)
A549	C	0,2 ± 0,1 (0,08 ± 0,04)
MCF7	C	0,3 ± 0,1 (0,13 ± 0,04)
HEK293T	NC	0,55 ± 0,03 (0,23 ± 0,01)
VA13	NC	1,2 ± 0,2 (0,51 ± 0,08)

The cytotoxicity was assayed by the MTT test. The concentrations of amicoumacin A in the growth media that caused 50% growth inhibition or cell death (IC_50_) is presented in both μM and μg/ml scale.

^*^- ‘C’ – cancerous cell line; ‘NC’ – non-cancerous cell line.
