# Supplementary material for: Amicoumacin A induces cancer cell death by targeting the eukaryotic ribosome
Source: Sci Rep. 2016 Jun 14;6:27720. doi: 10.1038/srep27720 (PMC4906347; doi:10.1038/srep27720)
Supplement: Supplementary Information [file srep27720-s1.pdf]

Amicoumacin A induces cancer cells death by targeting the eukaryotic ribosome

Irina V. Prokhorova, Kseniya A. Akulich, Desislava S. Makeeva, Ilya A. Osterman, Dmitry A. Skvortsov, Petr V. Sergiev, Olga A. Dontsova, Gulnara Yusupova, Marat M. Yusupov, Sergey E. Dmitriev

SUPPLEMENTARY FIGURES

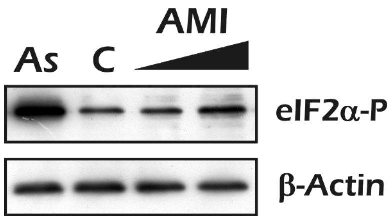

Supplementary Figure 1. Induction of eIF2 phosphorylation in HEK293T cells by amicoumacin A. C – untreated cells; As – 2 h incubation with 200 µM sodium arsenite; AMI – 2 h incubation with 1 µM or 5 µM amicoumacin A.

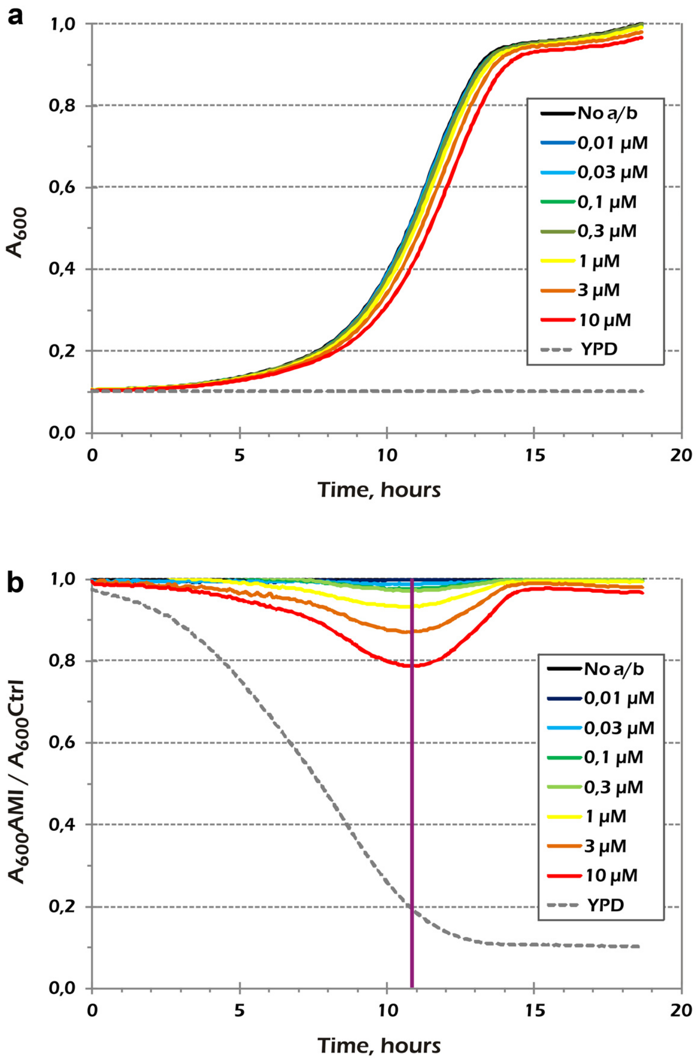

Supplementary Figure 2. Yeast culture growth curves. Yeast cells were grown in a microplate reader (in 24-well plate) at 30 °C with continuous shaking with or without amicoumacin A. (**a**) OD600 values measured every 5 min are shown. (**b**) The ratios of the OD600 values in amicoumacin A containing wells and antibiotic-free ones were calculated.

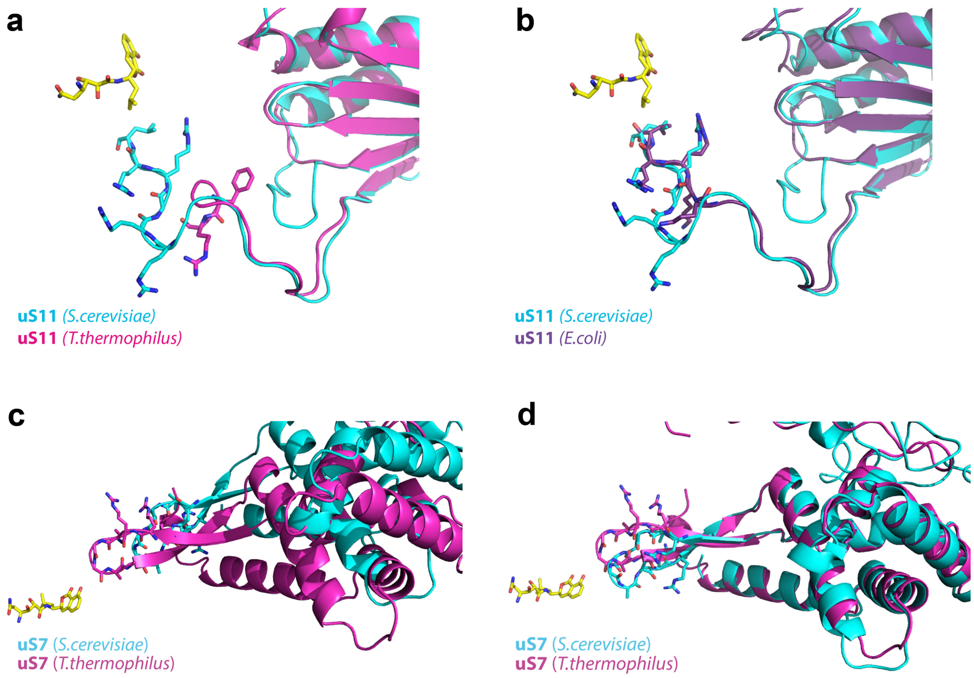

Supplementary Figure 3. Interactions of uS11 and uS7 proteins in eukaryotic and bacterial ribosome with amicoumacin A. The antibiotic is shown in yellow. (**a-c**) Structures of 80S and 70S ribosomes were aligned based on the helix 23 in 18S or 16S rRNA. (**a**) Yeast uS11 protein is shown in blue, uS11 from *T.thermophilus* is shown in pink (PDB entry 4W2F), and other ribosomal components are omitted for clarity. (**b**) Yeast uS11 protein is shown in blue, and uS11 from the structure of *E.coli* ribosome is shown in violet (PDB entry 4YBB). (**c**) Yeast uS7 is shown in blue, and uS7 from *T.thermophilus* is shown in pink (PDB entry 4W2F). (**d**) Alignment of the structure of uS7 protein from yeast ribosome on the structure of uS7 in bacterial ribosome. Amicoumacin A is shown in yellow, yeast uS7 is shown in blue, uS7 from *T.thermophilus* is shown in pink (PDB entry 4W2F), and other ribosomal components are omitted for clarity.

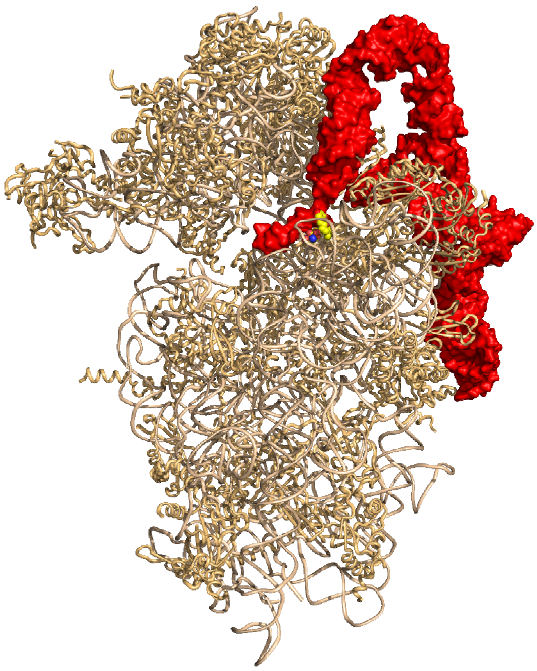

Supplementary Figure 4. Alignment of the structure of yeast 40S ribosomal subunit in complex with amicoumacin A and the structure of human 40S subunit in complex with HCV IRES (PDB entry 5A2G). Yeast 40S subunit is shown in wheat, amicoumacin A in yellow, HCV IRES in red, and the human 40S subunit is omitted for clarity.
